# gosset: An R package for analysis and synthesis of ranking data in agricultural experimentation

**DOI:** 10.1016/j.softx.2023.101402

**Published:** 2023-05

**Authors:** Kauê de Sousa, David Brown, Jonathan Steinke, Jacob van Etten

**Affiliations:** a Department of Agricultural Sciences, Inland Norway University of Applied Sciences, 2318 Hamar, Norway; b Digital Inclusion, Bioversity International, Parc Scientifique Agropolis II, 34397, Montpellier Cedex 5, France; c Laboratory of Geo-Information Science and Remote Sensing, Wageningen University & Research, Droevendaalsesteeg 3, 6708 PB, Wageningen, The Netherlands; d Digital Inclusion, Bioversity International, 30501, Turrialba, Costa Rica; e College of Agriculture and Life Sciences, Cornell University, 14853 Ithaca, NY, United States of America; f Thaer Institute of Agricultural and Horticultural Sciences, Humboldt University Berlin, Unter den Linden 6, 10099 Berlin, Germany

**Keywords:** Bradley–Terry, Data science, Plackett–Luce, On-farm trials, Tricot approach

## Abstract

To derive insights from data, researchers working on agricultural experiments need appropriate data management and analysis tools. To ensure that workflows are reproducible and can be applied on a routine basis, programmatic tools are needed. Such tools are increasingly necessary for rank-based data, a type of data that is generated in on-farm experimentation and data synthesis exercises, among others. To address this need, we developed the R package *gosset*, which provides functionality for rank-based data and models. The *gosset* package facilitates data preparation, modeling and results presentation stages. It introduces novel functions not available in existing R packages for analyzing ranking data. This paper demonstrates the package functionality using the case study of a decentralized on-farm trial of common bean (*Phaseolus vulgaris* L.) varieties in Nicaragua.

Code metadata

**Table:** 

Current code version	1.0
Code repository	https://github.com/ElsevierSoftwareX/SOFTX-D-22-00199
Legal code license	MIT
Code versioning system used	git
Software code languages, tools, and services used	R
Compilation requirements, operating environments & dependencies	R
Link to developer documentation	https://agrdatasci.github.io/gosset/
Support email for questions	desousa.kaue@gmail.com

## 1 Motivation and significance

Participatory on-farm experimentation approaches are reaching scale in agricultural research [1]. Participatory experiments often collect data as rankings, a format that is less common in other agricultural research settings [2]. A recently developed approach for on-farm experimentation, triadic comparison of technology options (tricot), makes intensive use of data in ranking format [3] and has already generated substantial trial datasets obtained from thousands of participating farmers [4], [5], [6], [7]. Also, a newly proposed approach for synthesizing crop variety evaluation data depends on the analysis of ranking data [8].

The analysis of ranking data requires the use of appropriate statistical models such as the Plackett–Luce model [9], [10] or the Bradley–Terry model [11]. Functionality for fitting these models is available in R with the packages *PlackettLuce* [12], *BradleyTerry2* [13] and *psychotree* [14]. However, extended functionality was required for the entire data science workflow, which usually includes: (1) Data preparation and cleaning, (2) modeling and validation, and (3) results presentation. For (1) *gosset* provides functions for converting and preparing data into a ranking or pairwise format required by the packages *PlackettLuce*, *BradleyTerry2* and *psychotree*. For (2), *gosset* provides functions for model selection and validation using cross-validation. In the case of (3), enhanced functionality for plotting model results is provided by the *gosset* package.

## 2 Software description

The R package *gosset* provides functionality supporting the analysis workflows in agricultural experimentation, especially for rank-based approaches. The package is available in the Comprehensive R Archive Network (CRAN) [15] and can be installed by executing install.packages(‘‘gosset’’). The package is named in honor of William Sealy Gosset, known by the pen name ‘Student’. Gosset was a pioneer of modern statistics in small sample experimental design and analysis. As a beer brewer at Guinness, he developed practical approaches to experimentation to compare barley varieties and beer brewing practices [16].

## 3 Software architecture

The R package *gosset* is structured following the guidelines described in the manual for creating R add-on packages [15]. This structure consists of files DESCRIPTION, LICENSE, NAMESPACE and NEWS, and directories data, dev, docs, inst, man, R, and vignettes. The package functions were developed following the S3 methods style and are contained in the R sub-directory.

## 4 Software functionalities

### 4.1 Data management and preparation

Ranking data comes in many different formats. For example, the tricot format consists in a ranking of three items as answers to two questions about the extremes of the ranking (i.e. best and worst). Other data come as numeric rankings. To be able to use these data, they need to be converted in formats that can be used by the model approaches. The *BradleyTerry2* and *psychotree* packages can deal with pairwise comparisons, while the *PlackettLuce* package can deal with rankings of several items.

• **rank_numeric** converts numeric values into rankings. The parameter ascending indicates if the rankings should be made considering the numeric values in ascending order. The default is ascending = FALSE. This function is useful when the data have been collected as numerical observations, for instance, in an experiment measuring crop yield.
• **rank_tricot** transforms data in tricot format into *PlackettLuce* rankings [12].
• **set_binomialfreq** transforms a PlackettLuce ranking object into binomial frequencies, as required by package *BradleyTerry2* [13].
• **set_paircomp** transforms a PlackettLuce ranking object into pairwise comparisons for BradleyTerry trees [14].

### 4.2 Modeling

The *gosset* package complements the R packages *BradleyTerry2*, *psychotree* and *PlackettLuce*, which were designed from a statistical perspective. These packages lack some functionality to work within a more predictive framework. Specifically, they lack functionality to perform more complex variable selection to generalize models across time and space and to evaluate these models in flexible ways. Therefore, *gosset* contains the following functions.

• **AIC** computes the Akaike Information Criterion [17] for a Bradley–Terry model or a Plackett–Luce model.
• **btpermute** deviance-based forward variable selection [18] procedure for Bradley–Terry models.
• **crossvalidation** performs k-fold cross-validation, where k could be specified by the user. The default is 10-fold. Folds can be provided as a vector for a custom cross-validation, such as blocked cross-validation.
• **kendallTau** computes the Kendall-tau rank correlation [19] coefficient between two rankings with p-values.
• **kendallW** computes Kendall’s W (coefficient of concordance) among observed rankings and those predicted by the Plackett–Luce model [20].
• **pseudoR2** computes goodness-of-fit metrics, such as McFadden’s pseudo-R^2^ [21].

### 4.3 Visualization and presentation of results

Bradley–Terry and Plackett–Luce models produce (log-)worth values, which are estimated (log-)probabilities that item *i* beats all the other items {*j*, …, *n*} in the same set of items. Given the specific characteristics of these values, *gosset* contains tailored methods to process these values into metrics that aid decision-making and to visualize these worth values.

• **compare** is a visualization approach to compare two different measures or traits [22]. An alternative to linear correlation plots. For instance, in the evaluation of crop variety trials, it allows to compare overall appreciation against yield. Another example is comparing the agreement records from different observers, like yield estimation collected by a technician and by a farmer.
• **plot** provides a ggplot2 plot with improved aesthetics and a large number of customization options as an alternative to the S3 method plot.pltree() implemented by the *PlackettLuce* package, which provides a base R plot.
• **regret** computes the regret coefficients, the loss under the worst possible outcome; a common heuristic in risk assessment strategy [23].
• **reliability** computes the probability of a set of items outperforming a reference item; a common heuristic in plant breeding [24].
• **worth_bar** creates a bar plot of the estimated worth for each evaluated item.
• **worth_map** creates a heatmap plot of the estimated log-worth for all items considering each of the evaluated traits.

## 5 Illustrative example

To demonstrate the functionality of the *gosset* package, we use the nicabean dataset, which was generated with decentralized on-farm trials of common bean (*Phaseolus vulgaris* L.) varieties in Nicaragua over five seasons (between 2015 and 2016). Following the tricot approach [3], farmers were asked to test in their farms three varieties of common bean. The varieties were randomly assigned as incomplete blocks, each representing 3 varieties out of a total set of 10 varieties. Each farmer assessed which of the three varieties in one incomplete block had the best and worst performance in eight traits (vigor, architecture, resistance to pests, resistance to diseases, tolerance to drought, yield, marketability, and taste). The farmers also provided their overall appreciation of the varieties, by indicating which variety had the best and the worst performance based on the overall performance considering all the traits. To analyze the data, we use the Plackett–Luce model implemented in the R package *PlackettLuce* [12].

The nicabean dataset is a list with two data frames. The first, trial, contains the trial data with farmers’ evaluations, ranked from 1 to 3, with 1 being the higher ranked variety and 3 the lowest ranked variety for the given trait and incomplete block. The rankings in this dataset were previously transformed from tricot rankings (where participants indicate best and worst) to ordinal rankings using the function rank_tricot(). The second data frame, covar, contains the covariates associated with the on-farm trial plots and farmers. This example will require the packages *PlackettLuce* [12], *climatrends* [25], *chirps* [26] and *ggplot2* [27].

**Figure:**
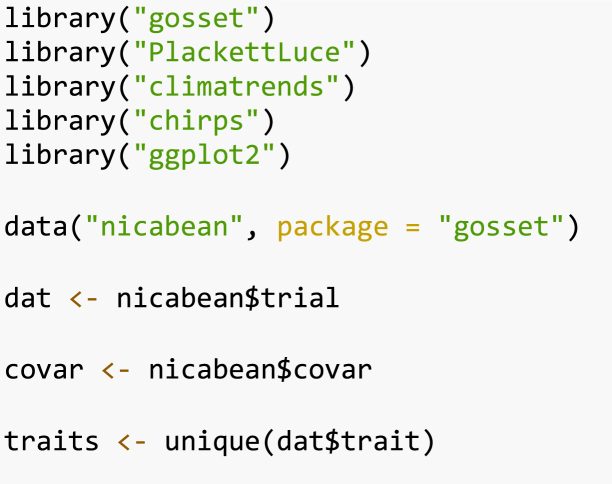

To start the data analysis, we transform the ordinal rankings into the Plackett–Luce​ rankings format (a sparse matrix) using the function rank_numeric We run iteratively over the traits adding the rankings to a list called R. Since the varieties are ranked in an ascending order, with 1 being the higher ranked and 3 the lower ranked, we use the argument ascending = TRUE to indicate which order should be used.

**Figure:**
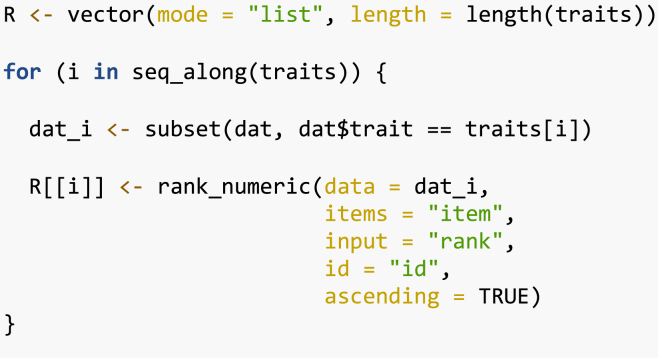

Then, using the function kendallTau() we assess the Kendall tau (τ) coefficient [19]. This approach can be used, for example, to assess what traits influence farmers’ choices or to prioritize traits to be tested in a next stage of tricot trials (e.g. a lighter version of tricot with no more than 4 traits to assess). We use the overall appreciation as the reference trait and compare the Kendall tau with the other 8 traits.

**Figure:**
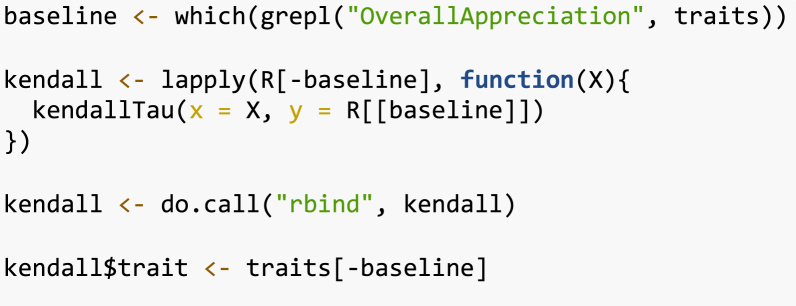

The Kendall correlation (Table 1) shows that farmers prioritized the traits yield (τ=0.749), taste (τ=0.653) and marketability (τ=0.639) when assessing overall appreciation.

Then, for each trait, we fit a Plackett–Luce model using the function PlackettLuce() from the package of the same name. This will allow us to continue the trial data analysis using the other functions in the package *gosset*.

**Table 1:** Kendall tau correlation between ‘overall performance’ and the other traits assessed in the Nicaragua bean on-farm trials.

Trait	kendallTau	Z value	Pr(>|z|)
Vigor	0.439	4.878	5.36e−07
Architecture	0.393	4.372	6.15e−06
Resistance To Pests	0.463	5.144	1.34e−07
Resistance To Diseases	0.449	4.998	2.90e−07
Tolerance To Drought	0.411	4.572	2.42e−06
Yield	0.749	8.325	4.22e−17
Marketability	0.639	7.100	6.22e−13
Taste	0.653	7.261	1.93e−13

**Figure:**
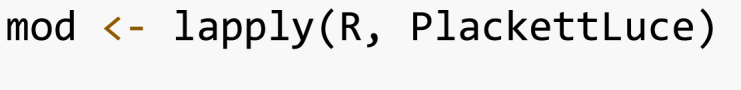

**Fig. 1:**
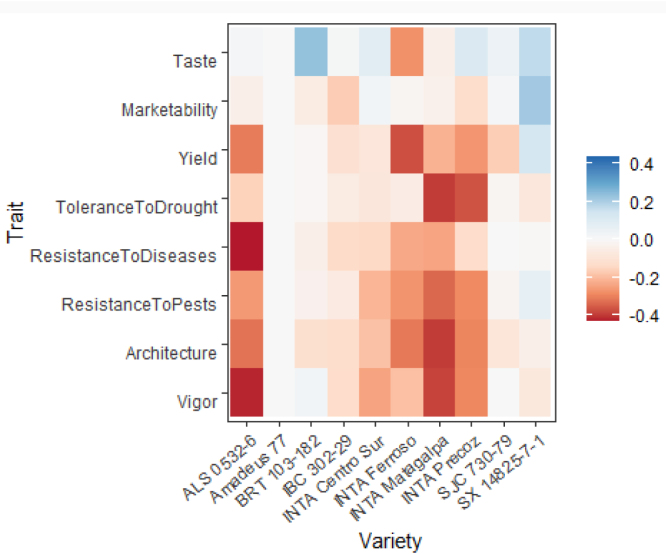
Trait performance (log-worth) of bean varieties in Nicaragua. Variety ‘Amadeus’ is set as reference (log-worth = 0). Blue values indicate a superior performance of varieties for a given trait, compared to the reference. Red values indicate a variety with weak performance for the given trait, compared to the reference.. (For interpretation of the references to color in this figure legend, the reader is referred to the web version of this article.)

The worth_map() function can be used to visually assess and compare item performance based on different characteristics. The values represented in a worth_map (Fig. 1) are log-worth estimates. From the breeder or product developer perspective the function worth_map() offers a visualization tool to help in identifying item performance based on different characteristics and select crossing materials.

**Figure:**
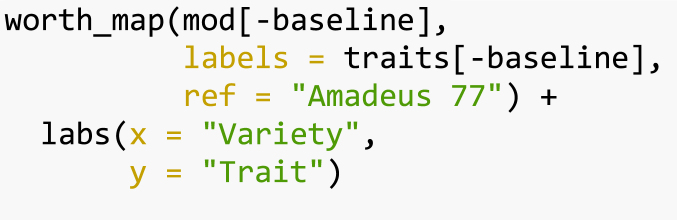

To consider the effect of climate factors on yield, we use agro-climatic covariates to fit a Plackett–Luce tree. For simplicity, we use the total rainfall (Rtotal) derived from CHIRPS data [28], obtained using the R package *chirps* [26]. Additional covariates can be used in a Plackett–Luce tree, for example using temperature data from R packages *ag5Tools* [29] or *nasapower* [30].

We request the CHIRPS data using the R package *chirps*. Data should be returned as a matrix. This process can take some minutes to be implemented.

**Figure:**
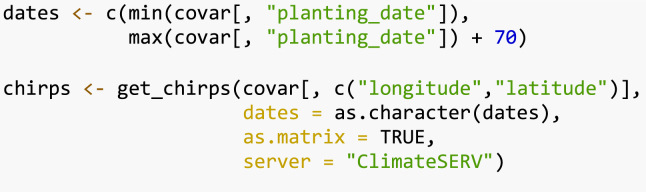

We compute the rainfall indices from planting date to the first 45 days of plant growth using the function rainfall() from the R package *climatrends* [25].

**Figure:**
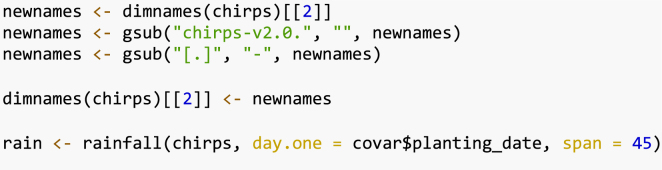

To be linked to covariates, the rankings should be coerced to a ‘grouped_rankings’ object. For this we use the function group() from *PlackettLuce*. We retain the ranking corresponding to yield.

**Figure:**
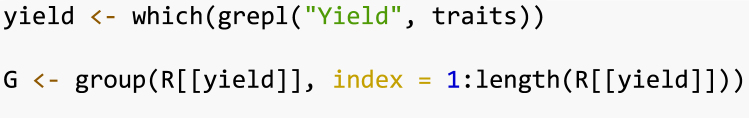

Now we can fit the Plackett–Luce tree with climate covariates.

**Figure:**
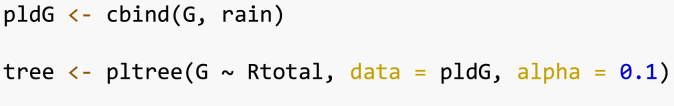

**Fig. 2:**
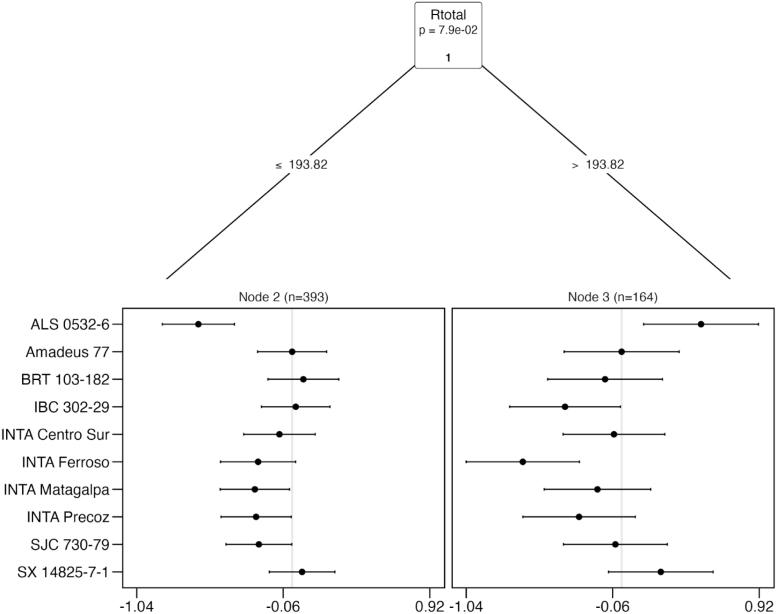
Effect of total rainfall (Rtotal) on yield of common beans in on-farm trials. Agroclimate variables are obtained from planting date over the first 45 days of plant growth. The x axis presents log-worth, the log-probability of outperforming the other varieties in the set.

The following is an example of the plot (Fig. 2) made with the function plot() in the *gosset* package. The functions node_labels(), node_rules() and top_items() can be used to identify the splitting variables in the tree, the rules used to split the tree and the best items in each node, respectively.

**Figure:**
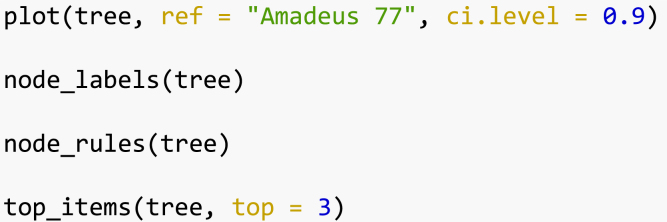

We can use the function reliability() to compute the reliability of the evaluated common bean varieties in each of the resulting nodes of the Plackett–Luce tree (Table 2). This helps in identifying the varieties with higher probability of outperforming a check variety (Amadeus 77). For the sake of simplicity, we present only the varieties with reliability ≥ 0.5.

**Figure:**
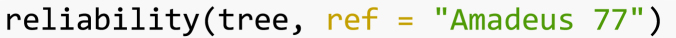

The results show that three varieties can marginally outperform Amadeus 77 under drier growing conditions (Rtotal ≤ 193.82 mm) whereas two varieties have a superior yield performance when under higher rainfall conditions (Rtotal > 193.82 mm) compared to the reference. This approach helps in identifying superior varieties for different target population environments. For example, the variety ALS 0532-6 shows weak performance in the whole yield ranking, however for the sub-group of higher rainfall, the variety outperforms all the others. Combining rankings with socio-economic covariates could also support the identification of superior materials for different market segments.

**Table 2:** Reliability of common bean varieties based on yield performance under different rainfall conditions from planting date to the first 45 days of plant growth. Variety Amadeus 77 is set as reference.

Node	Item	Reliability	ReliabilitySE	Worth
2	Amadeus 77	0.500	0.035	0.114
2	BRT 103-182	0.519	0.036	0.123
2	IBC 302-29	0.506	0.035	0.117
2	SX 14825-7-1	0.517	0.033	0.122
3	ALS 0532-6	0.630	0.056	0.177
3	Amadeus 77	0.500	0.058	0.104
3	SX 14825-7-1	0.565	0.053	0.135

A better approach for assessing the performance of varieties can be using the “Overall Appreciation”, since we expect this trait to capture the performance of the variety not only for yield, but for all the other traits prioritized by farmers (Table 1). To assess this, we use the function compare() which applies the approach proposed by Bland and Altman (1986) [22] to assess the agreement between two different measures. We compare overall appreciation vs yield. If both measures completely agree, all the varieties should be centered to 0 in the axis Y.

**Figure:**
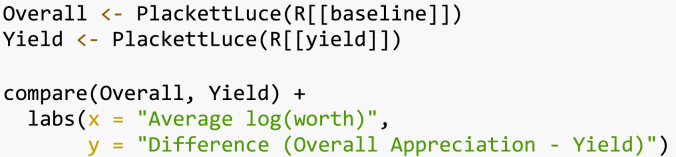

**Fig. 3:**
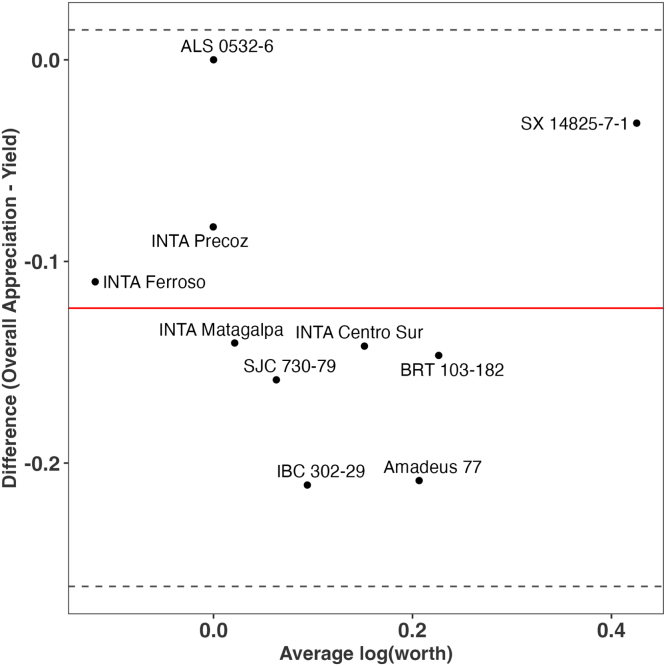
Agreement between overall appreciation and yield for crop variety performance in on-farm trials.

The chart (Fig. 3) shows no complete agreement between overall appreciation and yield. For example, variety SX 14825-7-1 shows superior performance for overall appreciation when compared with yield. Looking at the log-worth in the heat map of Fig. 1, we can argue that the superior performance of the given variety is also related to taste, marketability and disease resistance. This performance, however, was not captured when assessing only yield.

Here we present a simple workflow to assess crop variety performance and trait prioritization in decentralized on-farm trials with the tricot approach. Next steps in this workflow could also utilize other functions available in *gosset*, Examples include: (1) a forward selection combined with crossvalidation() to ensure model robustness, (2) model selection with btpermute() to consider all possible permutations in Bradley–Terry models, (3) a risk analysis using regret() to support the selection of varieties, and (4) using rank_numeric() to combine legacy data and deal with heterogeneous data from different trials. All of these were previously implemented and validated elsewhere [4], [5], [6], [7], [31], [32], [33].

## 6 Impact

Reproducible and efficient workflows are fundamental in scientific research [34]. The *gosset* package provides functionality that was not previously available from other R packages and which enabled scientific studies based on the analysis of ranking data. This functionality allows reproducibility and greater efficiency of the entire workflow. The utility of the *gosset* package has been demonstrated by enabling studies based on the analysis of decentralized on-farm trial data and/or heterogeneous data from different sources. For instance, van Etten et al. (2019) [4], Moyo et al. (2021) [5], de Sousa et al. (2021) [35], Brown et al. (2022) [7], Alamu et al. (2023) [6], Gesesse et al. (2023) [31] and Rutsaert et al. (2023) [33] applied the Plackett–Luce model in combination with recursive partitioning [12], [36]. In these studies, the *gosset* package supported data preparation, model validation and results presentation tasks. Furthermore, the *gosset* package is part of a software ecosystem built around ClimMob (https://climmob.net/), a digital platform for supporting on-farm trial management, which runs trials in more than 10,000 farms per year. Insights generated with the package’s functionalities are currently supporting several plant breeding teams in Sub-saharan Africa to select and advance breeding materials [6], [33]. Therefore, the *gosset* package is fundamental in the implementation of large scale on-farm experimentation projects. Refinement of methods and expansion of the approach in breeding programs is supported by an Africa-wide on-farm trial network implemented by the 1000FARMS Platform (https://1000farms.net/).

## 7 Conclusions

The use of ranking data in agricultural experimentation is currently growing, requiring new appropriate tools supporting analysis and synthesis activities. We developed the R package *gosset* to support the synthesis and analysis of ranking data, especially in agricultural research. The package provides functions that are not available in existing R packages for analyzing ranking data. This provides a friendlier user environment, streamlining the application of data science in agricultural research. In addition, the package code is open source, making it easier for developers to contribute but also to users to request new functionalities. We provided an illustrative example covering the main functionality across the stages involved in the analysis workflow. Since the package is also part of a growing community of practice in on-farm experimentation, it is expected that its functionality will be improved and expanded, pushed by the members of this community of practice.

## Declaration of Competing Interest

The authors declare that they have no known competing financial interests or personal relationships that could have appeared to influence the work reported in this paper.

## Data Availability

Data is freely available within the software
